# Mitogenomic analysis of a 50-generation chicken pedigree reveals a rapid rate of mitochondrial evolution and evidence for paternal mtDNA inheritance

**DOI:** 10.1098/rsbl.2015.0561

**Published:** 2015-10

**Authors:** Michelle Alexander, Simon Y. W. Ho, Martyna Molak, Ross Barnett, Örjan Carlborg, Ben Dorshorst, Christa Honaker, Francois Besnier, Per Wahlberg, Keith Dobney, Paul Siegel, Leif Andersson, Greger Larson

**Affiliations:** 1 BioArCh Biology S Block, University of York, Wentworth Way, Heslington, York YO10 5DD, UK; 2 Department of Archaeology, School of Geosciences, University of Aberdeen, St. Mary's, Elphinstone Road, AB24 3UF, UK; 3 School of Biological Sciences, University of Sydney, Sydney, New South Wales 2006, Australia; 4 Museum and Institute of Zoology, Polish Academy of Sciences, Warsaw 00-679, Poland; 5 Palaeogenomics and Bio-Archaeology Research Network, Research Laboratory for Archaeology, Dyson Perrins Building, South Parks Road, Oxford OX1 3QY, UK; 6 Department of Clinical Sciences, Swedish University of Agricultural Sciences, PO Box 7078, 75007 Uppsala, Sweden; 7 Science for Life Laboratory, Department of Medical Biochemistry and Microbiology, Uppsala University, PO Box 582, 75123 Uppsala, Sweden; 8 Department of Animal and Poultry Sciences, Virginia Tech, Blacksburg, VA 24061, USA; 9 Section of Population Genetics, Institute of Marine Research, Nordnes 5817, Bergen, Norway; 10 Department of Animal Breeding and Genetics, Swedish University of Agricultural Sciences, PO Box 7023, 75007 Uppsala, Sweden

**Keywords:** mitochondrial genome, pedigree, mutation rates, paternal leakage, association analysis

## Abstract

Mitochondrial genomes represent a valuable source of data for evolutionary research, but studies of their short-term evolution have typically been limited to invertebrates, humans and laboratory organisms. Here we present a detailed study of 12 mitochondrial genomes that span a total of 385 transmissions in a well-documented 50-generation pedigree in which two lineages of chickens were selected for low and high juvenile body weight. These data allowed us to test the hypothesis of time-dependent evolutionary rates and the assumption of strict maternal mitochondrial transmission, and to investigate the role of mitochondrial mutations in determining phenotype. The identification of a non-synonymous mutation in *ND4L* and a synonymous mutation in *CYTB*, both novel mutations in *Gallus*, allowed us to estimate a molecular rate of 3.13 × 10^−7^ mutations/site/year (95% confidence interval 3.75 × 10^−8^–1.12 × 10^−6^). This is substantially higher than avian rate estimates based upon fossil calibrations. Ascertaining which of the two novel mutations was present in an additional 49 individuals also revealed an instance of paternal inheritance of mtDNA. Lastly, an association analysis demonstrated that neither of the point mutations was strongly associated with the phenotypic differences between the two selection lines. Together, these observations reveal the highly dynamic nature of mitochondrial evolution over short time periods.

## 1. Introduction

Mitochondrial genomes have been widely used in biological research, especially when studying evolutionary and demographic processes that occur over short timeframes [1]. In vertebrates, mitochondrial evolution is characterized by strictly maternal inheritance and lack of recombination. Although various studies have suggested a constant rate of mitochondrial evolution among lineages and through time [2], there is now considerable evidence of a disparity between short- and long-term estimates of mitochondrial substitution rates [3–5]. Among the possible explanations for this pattern is that mitochondrial DNA (mtDNA) evolves non-neutrally, such that purifying selection removes negative mutations over time [6]. This naturally produces a pattern in which transient, deleterious mutations cause a short-term elevation in rates.

There have been few studies of short-term mitochondrial evolution, including both mutation rates and paternal leakage, particularly in non-human vertebrates [7,8]. Estimates of mitogenomic mutation rates have been obtained in studies of Adélie penguins [6,9] and humans [10] and these rates greatly exceed those inferred from longer phylogenetic timescales. Evidence for paternal inheritance of mtDNA (and other ‘rare’ evolutionary phenomena) is accumulating in multiple species, including humans [11] and sheep [12], but it is usually only visible in laboratory or controlled conditions [13–15]. As a result, its frequency may be underappreciated. This is compounded by the assumption that in natural populations, without direct knowledge of genetic relatedness and transmission, all mtDNA is maternally inherited. Combined with the low power associated with standard detection methodologies, it has been difficult to assess rates of paternal leakage in natural populations [13].

Domesticated animals present ideal systems for studying mitochondrial evolution in vertebrates, particularly if they have documented pedigrees. One such pedigree has been recorded for the Virginia chicken lines, an experimental White Plymouth Rock population spanning more than 50 generations. This pedigree, initiated in a founder population of seven partially inbred lines, was subjected to annual divergent selection for high and low body-weights at 56 days of age. This approach established high (HWS) and low (LWS) weight selected lines that now possess a greater than 10-fold difference in body weight at selection age [16–18].

Here, we used this well-documented chicken pedigree to perform a detailed investigation of short-term mitochondrial evolution in a vertebrate system. More specifically, we estimated the mitochondrial mutation rate, tested for instances of non-maternal inheritance, and examined the degree to which mitochondrial mutations were responsible for the divergent phenotypes of the two selected lines.

## 2. Material and methods

**Figure 1.**
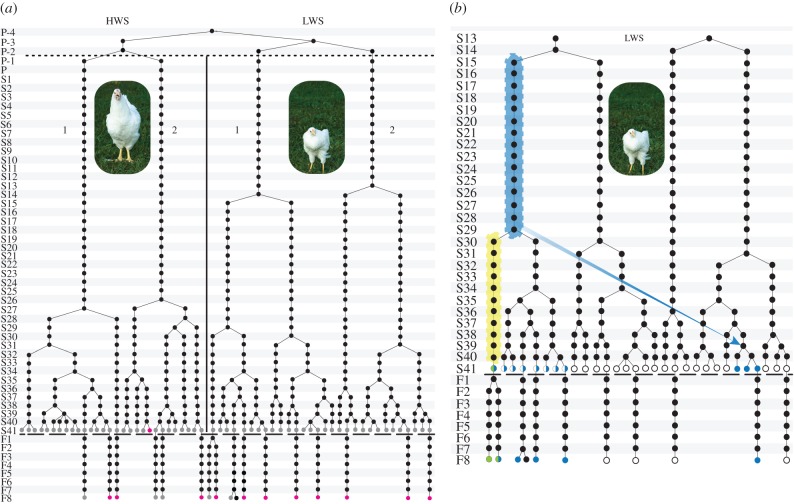
Chicken pedigree from which mitochondrial genomes were sequenced. (*a*) Overview of the maternal lineages of the chicken pedigree, comprising high weight selected (HWS) and low weight selected (LWS) lines. Pink circles indicate individuals from which we sequenced complete mitochondrial genomes and grey circles represent those that were typed for the mutations in *CYTB* and *ND4L*. Black circles indicate individuals that were either not sampled or not successfully sampled. Codes on the left-hand side refer to generations before (P) and after (S) the selection experiment began, and following the initiation of the inter-cross experiments (F). The numerals 1 and 2 level with the chicken figures refer to the two maternal lines present in the HWS and LWS, respectively. (*b*) Subset of the pedigree from S13 to F_8_ and additional detail of the LWS line. Blue and yellow shading indicates the timing and lineage on which the *ND4L* and *CYTB* mutations occurred on the pedigree, respectively. Genotyped individuals that possessed the *ND4L* mutation are shown in blue and those that were heteroplasmic for *ND4L* are shown in white and blue. Those that possessed both mutations but were heteroplasmic for the *CYTB* mutation are shown in green and blue, the individual that was homoplasmic for both mutations is shown in green. Those that were tested but possessed neither mutation are shown in white. The blue arrow represents the instance of paternal leakage. It starts on the lineage from which the male involved in the paternal leakage was derived, and points to the female whose offspring inherited the male's mitochondrial genome. Further details are in the electronic supplementary material.

We identified and sequenced the mitogenomes of the 12 most distantly related individuals on the maternal pedigree, representing 385 mitochondrial transmissions. This sampling scheme provided an efficient means of capturing a large number of mitochondrial transmissions with a limited sample of mitogenomes (figure 1*a*). We used multiple overlapping PCR and Sanger sequencing primer pairs (electronic supplementary material, table S2) and aligned the resulting genomes using CodonCode (http://www.codoncode.com. CodonCode Corporation).

The single-nucleotide polymorphisms (SNPs) detected in the *ND4L* and *CYTB* genes were genotyped using DNA isolated from blood (electronic supplementary material). In order to establish potential heteroplasmy, we carried out pyrosequencing of the 12 original individuals and of an additional 66 chickens from generation S41, the most recent generation of the pedigree and the F_8_ generation of a deep inter-crossed population of the two selection lines (figure 1*a*; electronic supplementary material, table S4). The base for the inter-cross line was reciprocal parent line and F_1_ crosses (electronic supplementary material). An association analysis was carried out using birds from the F_8_ generation to explore the possible link between these mitochondrial mutations in the LWS and the marked phenotypic differences between HWS and LWS chickens.

The rate of evolution was calculated by taking into account the number of observed mutations in the approximately 16 000 bp mitochondrial genome over 47 years and 385 transmissions. Uncertainty in the estimate was calculated using the binomial confidence interval.

## 3. Results and discussion

The reconstruction of the maternal pedigree based on the mitogenome sequences allowed us to identify two separate point mutations and an instance of paternal leakage, all of which occurred in the LWS line (figure 1*b*). The first mutation, a non-synonymous G–A transition in *ND4L*, occurred between generations S15 and S29 on branch 1. The most likely explanation for the presence of this mutation in LWS branch 2 (figure 1*b*) is an instance of paternal leakage that took place in generation S39 (electronic supplementary material). A second mutation, a synonymous A–G transition in the *CYTB* gene, occurred between generations S30 and S40 in an individual that already possessed the *ND4L* mutation. We found evidence for mtDNA heteroplasmy with subsequent fixation in these lines (figure 1*b*; electronic supplementary material), a common observation in maternal lineages after a new mtDNA mutation has occurred [19].

**Figure 2.**
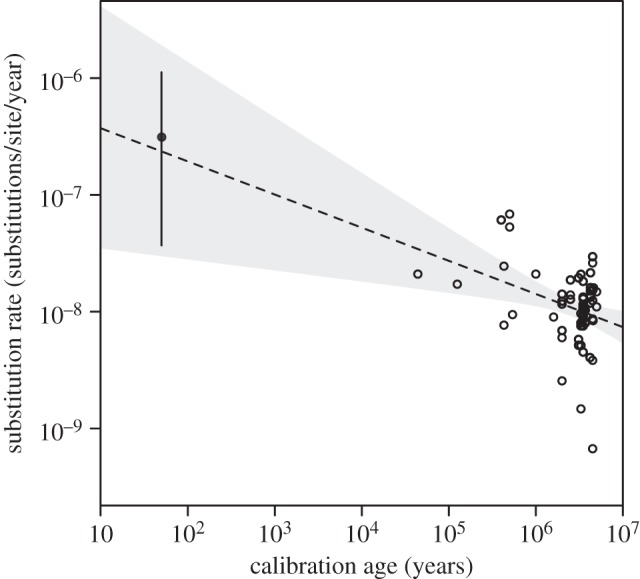
Relationship between published estimates of substitution rates and calibration age from 65 bird datasets (empty circles) using only coding mtDNA (adapted from [5]). The filled circle on the top left-hand side of the plot represents the pedigree estimate from this study and was not used to derive the regression line. Our calculation may be an underestimate given the potential for back mutations between the founding line and the sampled birds in generation S41, though this is unlikely. The dashed line is a regression trendline estimated solely from the 65 published rate estimates. Grey shading represents the 95% confidence interval of the trendline.

The presence of these two novel mutations allowed us to estimate a mutation rate of 3.13 × 10^−7^ mutations/site/year (95% confidence interval 3.75 × 10^−8^–1.12 × 10^−6^). Our estimate is consistent with an expectation of a higher rate estimate over shorter timescales, as demonstrated by the trendline resulting from a correlation between previously published avian rate estimates and the timescale over which they were estimated (figure 2). We observe this strong relationship despite evidence of substantial rate heterogeneity in birds, with synonymous substitution rates in mitochondria varying among taxa by more than a factor of 30 [20]. Our pedigree-based estimate of the mutation rate is consistent with the short-term elevation of rate estimates caused by the presence of transient mutations, a phenomenon that has been observed in pedigree studies of humans and other mammals [21]. Combined with previous evidence of a time-dependent pattern in rate estimates [5], this has important consequences for estimating the timescales of recent evolutionary events using molecular clocks [4].

Mapping the mutations onto the pedigree not only allowed us to establish when the mutations occurred, but also to identify a clear instance of paternal leakage in the LWS line (figure 1*b*). A subsequent investigation of the combined maternal and paternal records allowed us to identify the specific individuals in which the paternal leakage occurred (electronic supplementary material). This phenomenon is likely to be generally underappreciated given the difficulty in confidently recognizing the phenomenon in wild populations and the lack of sensitivity in detection methods. Our observation of an instance of paternal leakage in this pedigree suggests that this phenomenon might not be as rare as is commonly assumed.

The non-synonymous mutation at a first codon position in *ND4L* has, to our knowledge, not been previously reported in chickens, but another galliform*, Polyplectron germaini,* possesses the same nucleotide and amino acid (electronic supplementary material, figure S1). The second mutation (a synonymous change in *CYTB*) has been previously identified in other vertebrates (electronic supplementary material, figure S2).

Because the observed mutations occurred solely in the LWS line, they may have been partially responsible for the divergent phenotypes of the two selected lines. To investigate this, an association analysis was carried out to assess whether the two mitochondrial mutations had a major effect on body weight at hatch—and at 2, 4, 6, 8 and 10 weeks of age—that differentiated the two lines. A previous quantitative trail loci (QTL) analysis of the F_2_ generation suggested that phenotypic differences between reciprocal matings may have been caused by genetic variation in mtDNA [22]. Here, however, we found no significant effect between the presence of these mutations and growth traits in the F_8_ generation for either *CYTB* or *ND4L* (electronic supplementary material, table S5). As a result, these data suggest that neither of these mutations is significantly correlated with the extreme difference in early growth between the two lines.

Overall, our analysis of a long-term chicken pedigree has revealed the complex nature and dynamism of mitochondrial evolution when observed over evolutionarily short time periods. The observations of a rapid rate of evolution and an incidence of paternal leakage have several ramifications. First, molecular clock analyses often uncritically import evolutionary rates calculated using fossil calibrations. Our study provides further evidence that short-term rates can be much higher and that a failure to take this into account will lead to overestimation of the timeframe of recent evolutionary events. In addition, understanding the frequency of paternal inheritance of mtDNA is key to determining how and why different taxa maintain uniparental inheritance of mitochondria. Finally, our study provides a demonstration of the evolutionary insights that can be gleaned from detailed studies of well-documented animal pedigrees.

## Supplementary Material

Supplementary Information Text

## Supplementary Material

Supplementary Tables S1-S5

## Supplementary Material

Supplementary Figure S1

## Supplementary Material

Supplementary Figure S2

## Data accessibility

The 12 mitochondrial genomes sequenced as part of this project are available on GenBank, accessions KT626847–KT626858.
