# Supplementary material for: Mitogenomic analysis of a 50-generation chicken pedigree reveals a rapid rate of mitochondrial evolution and evidence for paternal mtDNA inheritance
Source: Biol Lett. 2015 Oct;11(10):20150561. doi: 10.1098/rsbl.2015.0561 (PMC4650172; doi:10.1098/rsbl.2015.0561)
Supplement: Supplementary Information Text [file rsbl20150561supp1.docx]

**Mitogenomic analysis of a 50-generation chicken pedigree reveals a rapid rate of mitochondrial evolution and evidence for paternal mtDNA inheritance**

**Supplementary Material**

**Detailed methodology**

The mitochondrial pedigree used in this study is a partial reconstruction of the Virginia Tech chicken pedigree based on relationships and inheritance patterns that trace back the female lines that contributed to S41 intercross individuals, following every individual’s mother back through each generation. The foundation stock for the selected lines consisted of crosses of 7 moderately inbred lines of White Plymouth Rock chickens that had been developed at Virginia Tech. From this segregating gene pool, 2 lines were selected on the basis of body weight at 56 days of age. Chickens with the heavier body weights formed the parent stock for the high weight line whereas those with the lighter body weights were used as parents for the low weight line. Thereafter, individual phenotypic selection was practiced within each line for the single trait, body weight at 56 days of age. Within each line, males and females were selected to reproduce the subsequent generations. Females were assigned to individual males with the only restriction being that no half sibs or matings of closer relationship were permitted. Through generation 4, matings consisting of 8 sires and 48 dams were used to produce each selected line. Generations 5 through 25 were produced from matings of 12 sires and 48 dams. Thereafter, matings involved 14 sires and 56 dams. Wright’s inbreeding coefficients calculated for each line in generation 48 was .30 and .26 in the high and low lines, respectively. Effective population size was 32.1 and 38.3 in the respective high and low lines with an effective number for founders being the same (15.7) in both lines. More specific details are in references [[1-5](#_ENREF_1)].

The intercross was conducted as follows. From the S41 generation of the HWS and LWS lines, a reciprocal cross was designed so that 10 HWS males were mated to 22 LWS females and 8 LWS males were mated to 19 HWS females. From the F_1_ generation, 8 males and 75 females were reciprocally inter-crossed to produce 874 F_2_ chickens. This was accomplished by mating each of 4 H x L males to 9 or 10 L x H females and 4 L x H males to 9 or 10 H x L females. This scheme precluded sib or half sib matings. The 874 individuals were from a single hatch with all parents of the same age. Thereafter, pair matings (1 male: 1 female) for subsequent generations were a restricted random to avoid matings between siblings. The number of pair matings approximated 40 per generation. Although this procedure avoided half and full sib matings, they did not preclude matings of cousins. Details are given in the references above.

A breakdown of the chicken identification numbers for the pedigree used in this study is given in table S1. The identification numbers for the chickens selected for full mtDNA genome analysis are 5456, 1670, 5349, 5315, 5210, 5326, 5332, 5600, 5280, 5298, 5192, 5216. Genomic DNA was isolated from blood samples in all cases from individuals on the same day at the same age (around 58-60 days old), thereby mitigating tissue specific and age related mtDNA heteroplasmy [[6](#_ENREF_6)].

Full mitochondrial genomes were amplified using conventional PCR. Primers were those used in previous publications [[7](#_ENREF_7), [8](#_ENREF_8)] that were aligned and some slightly modified to include degenerate positions. Resultant sequences were aligned by eye using CodonCode [[9](#_ENREF_9)] to a reference mitochondrial genome for chicken from the NCBI database under accession number NC_001323. Each SNP was verified using pyrosequencing, which was carried out using PyroMark Gold (Qiagen) reagents on a Pyro Mark Q96MD (Qiagen) instrument. PCR products were generated using KAPA2G Robust HotStart DNA Polymerase (Kapa Biosystems). The results for the proportion of the two SNPs read by the instrument were generated by the software and are available in table S4. PCR and sequencing primers are listed in table S2 and pyrosequencing primers can be found in table S3.

*Mutation characterization and mtDNA heteroplasmy*

The mitochondrial sequences suggested the following scenario. Two point mutations and an instance of paternal leakage occurred in the LWS alone. The first mutation, a G-A transition in the NADH dehydrogenase subunit 4L gene (*ND4L*) took place between generations S15 and S29 in branch 1 of the LWS, and was passed down to birds sequenced from the S41 generation in both major branches of the LWS. The same *ND4L* mutation was also detected in three chickens typed in the S41 generation of the other LWS2 lineage, and a direct F_8_ descendant. The male pedigree revealed that a male offspring of a female that possessed the mutation was the parent of a female whose maternal lineage led to the S41 female 1940, who has the mutation along with its close relatives 1945 and 2040. The female 1940 then passed on the mutation to offspring that formed one of the intercross lines leading to the F_8_ descendent who also carried the mutation.

Of the 17 chickens that possessed the *ND4L* SNP, pyrosequencing revealed that eight were heteroplasmic and of the three that possessed the *CYTB* SNP, two were heteroplasmic (table S4). Heretoplasmy in mtDNA often persists for multiple generations since offspring can inherit multiple copies of both the wild and mutated mtDNA before the mutation either reaches fixation or is eliminated [[9-12](#_ENREF_9)]. In this pedigree, the *ND4L* mutation nearly reached fixation by the F_8_ generation in the intercross.

BLAST searches were performed on the identified SNPs. The *ND4L* SNP change from G to A alters the amino acid sequence of the protein substituting alanine (A) for threonine (T). This leads to a decrease of hydrophobicity from 0.74 to 0.72 (protein information taken from GPMAW lite). The *ND4L* SNP does not exist among the *Gallus* mitochondrial sequences that have been submitted to Genbank, however, another galliform, *Polyplectron germaini,* possesses an A at this position and shares the same amino acid when the DNA sequence is translated (figure S1). There is also no *Gallus* sequence that possesses the *CYTB* mutation reported here. The *CYTB* SNP has, however, been identified in a range of other birds and vertebrates. Both the wild type (A) and mutation (G) are found amongst sequences of *CYTB* in dogs (figure S2).

*Detection of paternal inheritance*

We detected the same *ND4L* mutation in chickens from both the LWS1 and LWS2 lineages, despite the fact that these chickens were distantly related, and many more closely related chickens did not possess the mutation (figure 1*b*). This pattern suggests that either the mutation occurred independently, or that the LWS2 chickens acquired the mutation through the paternal line following an instance of paternal leakage. In order to distinguish between these two alternatives, we performed an analysis of the paternal inheritance patterns using hardcopy pedigree records to assess the status of the sires of the chickens in LWS2 that were shown to possess the mutation. Our results suggest that the mutation did not occur twice independently, but is instead an instance of paternal leakage.

The precise history is as follows. Given the pattern of the *ND4L* mutation in the low-weight line, it is reasonable to assume that female chicken 840c hatched in generation S29 possessed the mutation. This chicken had two clutches in S30: the first included daughter 575, located in LWS1 in on our mtDNA pedigree and this maternal line eventually led to female 1934 in S41 who possessed the *ND4L* mutation. The second clutch, which is not on our pedigree (because no females from this branch left female offspring in the S41 lineage), included the female 2065 that would have also had the mutation. The maternal line from 2065 leads to a male, 1353 hatched in generation S38 (not on our pedigree) who was mated with female 1503 found in LWS2 in our pedigree. Female 1503 did not have the mutation, as the maternal line from her female sibling 1501 does not lead to female carrying the mutation in S41. The mating between female 1503 and male 1353 produced the female offspring 2457 in generation S39. This is when the paternal inheritance must have occurred, leading to offspring that possessed the father's mitogenome that contained the mutation. From there, the maternal inheritance pattern of mtDNA resumes and the maternal line leading down from 2457 leads to female 1940 in generation S41, who also possessed the mutation.

The timing of this paternal inheritance of mtDNA in S39, accounts for why S41 females 2040, 1945 and 1940 have the mutation but 2108, 2104, 1997 and 2064 do not. This pattern also indicates that the *ND4L* mutation must have occurred at some point between generations S15 and S29, and that the branching took place before the *CYTB* mutation was layered on top of the *ND4L* mutation.

*Association analysis*

The association analysis was carried out to explore a possible link between mitochondrial mutations in the LWS and the marked phenotypic differences between HWS and LWS chickens. The analysis was carried out using a linear regression model using body weight, sex and mtDNA genotype as outlined in Table S5; mtDNA genotype was scored as the percentage of the mutant allele, since we observed heteroplasmy. We used birds from the F_8_ generation in an advanced intercross line established based on the HWS and LWS intercross. The reason to use the F_8_ generation rather than the F_2_ generation was to avoid confounding between mtDNA and maternal effects since only a limited number of the LWS founder females carried the mtDNA mutations. However, the major limitation in this analysis is the small number of birds carrying the mutations, only three for *CYTB* and seven for *ND4L*. Thus, we could only test for any major effects on growth of these mutations. Analysis of the F_8_ data did not reveal any statistically significant effects neither for *CYTB* nor for *ND4L*.

**Legends of additional tables and figures**

**Table S1**

A list of identifying chicken sample numbers by generation in the left to right order they are listed on Figure 1.

**Table S2**

A list of amplification and sequencing primers used to generate full mtDNA genomes in this study. Primer sequences were slightly modified (by including degenerate bases) or were ordered unmodified from previous studies [[7](#_ENREF_7), [8](#_ENREF_8)].

**Table S3**

A list of the primer sets used for pyrosequencing.

**Table S4**

Pyrosequencing results for chickens from the F_8_ and S41 generations included in this paper, indicating % of the wild type SNP ± 1σ.

**Table S5**

Effect of mtDNA genotype, scored as the percentage of the mutant sequence, as regards *de novo* mutations in *ND4L* and *CYTB* on body-weight at various ages in the F_8_ generation (n=378) from an intercross between Virginia High-Weight and Low-Weight chickens. The mtDNA genotype was determined by pyrosequencing or for many of the wild type birds deduced based on the maternal lineage.

**Figure S1**

Alignments of (a) 60bp of homologous DNA sequences surrounding the ND4L SNP reported in this study found in other bird taxa, taking those that were ≥90% identical on a BLAST search of the entire gene excluding *Gallus gallus* sequences; (b) similar alignments for vertebrate taxa, and (c) protein translations of ND4L for the wild and mutant chicken amino acid sequence including *P.germaini* which shares a T residue in the same position as the mutant type. Nucleotide and protein alignments were generated using Clustal Omega (EMBL-EBI).

**Figure S2**

Alignments of 60bp of homologous DNA sequences surrounding the CYTB SNP reported in this study found in (a) other bird taxa, taking those that were ≥90% identical on a BLAST search of the entire gene excluding *Gallus gallus* sequences; (b) vertebrate taxa. Nucleotide and protein alignments were generated using Clustal Omega (EMBL-EBI).

**References**

[1] Marquez, G., Siegel, P. & Lewis, R. 2010 Genetic diversity and population structure in lines of chickens divergently selected for high and low 8-week body weight. *Poultry Science* **89**, 2580-2588.

[2] Dunnington, E., Honaker, C., McGilliard, M. & Siegel, P. 2013 Phenotypic responses of chickens to long-term, bidirectional selection for juvenile body weight—Historical perspective. *Poultry Science* **92**, 1724-1734.

[3] Dunnington, E. & Siegel, P. 1996 Long-term divergent selection for eight-week body weight in White Plymouth Rock chickens. *Poultry Science* **75**, 1168-1179.

[4] Siegel, P.B. 1962 Selection for body weight at eight weeks of age 1. Short term response and heritabilities. *Poultry Science* **41**, 954-962.

[5] Siegel, P.B. 1962 A double selection experiment for body weight and breast angle at 8 weeks of age in chickens. *Genetics* **47**, 1313-1319.

[6] Jenuth, J.P., Peterson, A.C. & Shoubridge, E.A. 1997 Tissue-specific selection for different mtDNA genotypes in heteroplasmic mice. *Nature genetics* **16**, 93-95.

[7] Nishibori, M., Hayashi, T., Tsudzuki, M., Yamamoto, Y. & Yasue, H. 2001 Complete sequence of the Japanese quail (Coturnix japonica) mitochondrial genome and its genetic relationship with related species. *Animal genetics* **32**, 380-385.

[8] Nishibori, M., Hanazono, M., Yamamoto, Y., TSUDZUKI, M. & Yasue, H. 2003 Complete nucleotide sequence of mitochondrial DNA in White Leghorn and White Plymouth Rock chickens. *Animal Science Journal* **74**, 437-439.

[9] <http://www.codoncode.com>. (CodonCode Corporation.

[10] Laipis, P.J., Van De Walle, M.J. & Hauswirth, W.W. 1988 Unequal partitioning of bovine mitochondrial genotypes among siblings. *Proceedings of the National Academy of Sciences* **85**, 8107-8110.

[11] Millar, C.D., Dodd, A., Anderson, J., Gibb, G.C., Ritchie, P.A., Baroni, C., Woodhams, M.D., Hendy, M.D. & Lambert, D.M. 2008 Mutation and evolutionary rates in Adélie penguins from the Antarctic. *PLoS Genetics* **4**, e1000209.

[12] Rand, D.M. 2001 The units of selection of mitochondrial DNA. *Annual Review of Ecology and Systematics*, 415-448.
