# Supplementary material for: Mitogenomic analysis of a 50-generation chicken pedigree reveals a rapid rate of mitochondrial evolution and evidence for paternal mtDNA inheritance
Source: Biol Lett. 2015 Oct;11(10):20150561. doi: 10.1098/rsbl.2015.0561 (PMC4650172; doi:10.1098/rsbl.2015.0561)
Supplement: Supplementary Tables S1-S5 [file rsbl20150561supp2.docx]

**Table S1**

A list of identifying chicken sample numbers by generation in the left to right order they are listed on Figure 1.

| **Generation** | **Year** | **Chicken ID** |
| --- | --- | --- |
| P-1 |  | 112n, 112a, 623m, 117k |
| P |  | 827i, 820c, 902x, 874f |
| S1 | 1958 | 342l, 380i, 561e, 521a |
| S2 | 1959 | 400p, 380i, 623i, 601d |
| S3 | 1960 | 342o, 443o, 624i, 504o |
| S4 | 1961 | 360o, 464a, 607g, 561c |
| S5 | 1962 | 543o, 343d, 722j, 782v |
| S6 | 1963 | 546d, 560i, 664p, 743p |
| S7 | 1964 | 345b, 463c, 800d, 683j |
| S8 | 1965 | 480c, 383b, 744j, 600f |
| S9 | 1966 | 361d, 441e, 743k, 622l |
| S10 | 1967 | 302j, 543i, 665d, 741a |
| S11 | 1968 | 441j, 304b, 803h, 743c |
| S12 | 1969 | 543j, 481z, 583g, 683b |
| S13 | 1970 | 322g, 341f, 760a, 781a |
| S14 | 1971 | 300i, 541i, 763d, 723b, 723d |
| S15 | 1972 | 420l, 483d, 763c, 763e, 641e, 662g |
| S16 | 1973 | 502h, 421g, 640i, 603g, 660c, 760l |
| S17 | 1974 | 301e, 422d, 800k, 662f, 700f, 583d |
| S18 | 1975 | 340d, 461j, 624i, 761d, 642i, 700g |
| S19 | 1976 | 342i, 482c, 720g, 743g, 643d, 703h |
| S20 | 1977 | 300h, 422g, 682e, 643e, 800c, 681d |
| S21 | 1778 | 400b, 482g, 721h, 780d, 663a, 601d |
| S22 | 1979 | 541e, 402c, 601b, 642g, 782f, 600b |
| S23 | 1980 | 421l, 442n, 660a, 681d, 603d, 580f |
| S24 | 1981 | 382e, 303f, 641d, 761a, 760a, 580c |
| S25 | 1982 | 460b, 320l, 602c, 740j, 662c, 660b |
| S26 | 1983 | 503j, 461f, 683f, 640b, 741k, 682j |
| S27 | 1984 | 363c, 433i, 433l, 841a, 701c, 583, 822d |
| S28 | 1985 | 421e, 421i, 544b, 464b, 603c, 741g, 843f, 604a |
| S29 | 1986 | 421a, 401c, 301c, 540k, 540b, 840c, 601a, 701c, 780e |
| S30 | 1987 | 757, 2145, 731, 785, 2174, 2148, 576, 575, 470, 606, 680 |
| S31 | 1988 | 95, 80, 112, 50, 89, 160, 484, 241, 475, 478, 500, 438 |
| S32 | 1989 | 1776, 2219, 2267, 2260, 1763, 1737, 1780, 2167, 1917, 2110, 1974, 2059, 1909, 1910 |
| S33 | 1990 | 2036, 2059, 2017, 2231, 2067, 2194, 2053, 2305, 2427, 2510, 2448, 2494, 2402, 2321 |
| S34 | 1991 | 5006, 4916, 4870, 4925, 4943, 4897, 5080, 5168, 5177, 5319, 5255, 5122, 5148 |
| S35 | 1992 | 1644, 1698, 1693, 1623, 1633, 1625, 1512, 1501, 1490, 1808, 1873, 1880, 1770, 1968, 1963, 1972, 1858, 1987 |
| S36 | 1993 | 261, 122, 339, 192, 315, 134, 139, 242, 356, 96, 667, 629, 630, 493, 361, 478, 400, 465, 548, 550, 582 |
| S37 | 1994 | 663, 751, 518 , 517, 589, 656, 504, 739, 695, 625, 658, 994, 1050, 817, 909, 1063, 1032, 944, 895, 898, 933, 969, 838 |
| S38 | 1995 | 1179, 1210, 1283, 1116, 1078, 1134, 1266, 1153, 1194, 1124, 1171, 1313, 1375, 1367, 1368, 1486, 1488, 1498, 1477, 1516, 1513, 1416, 1537, 1475, 1501, 1503, 1447 |
| S39 | 1996 | 2418, 2372, 2375, 2359, 2332, 2383, 2411, 2263, 2282, 2366, 2364, 2232, 2319, 2487, 2562, 2536, 2543, 2436, 2505, 2528, 2476, 2478, 2493, 2509, 2581, 2446, 2471, 2445, 2457, 2431, 2430 |
| S40 | 1997 | 4014, 2522, 4071,4344, 2552, 4065, 2509, 2531, 4303, 4334, 4320, 4010, 4033, 4038, 4329, 2541, 4077, 4015, 4204, 2712, 2713, 4184, 4119, 4114, 2603, 2694, 4113, 4123, 4192, 2635, 2636, 2653, 2664, 2588, 4104, 2630, 2626, 4142, 4219, 4216, 4199, 2648, 2650 |
| S41 | 1998 | 1653, 1654, 1655, 1822, 1819, 1774, 1712, 1844, 1833, 1832, 1757, 1740, 1736, 1812, 1815, 1720, 1678, 1680, 1890, 1674, 1670, 1897, 1896, 1728, 1690, 1786, 1784, 1871, 1879, 2080, 2809, 2068, 2130, 2026, 1959, 1937, 1934, 1932, 1992, 2029, 2012, 1988, 1926, 1927, 1925, 2132, 1953, 2048, 2100, 2108, 2104, 1997, 2064, 2040, 1945, 1940, 1967, 1972, 2109 |
| F1 |  | 153, 195, 145 , 210, 186, 170, 241, 219, 301, 318, 313, 294, 265, 249, 246 |
| F2 |  | 1428, 1424, 1412, 1402, 1468, 1435, 1378, 1379, 1349, 1386, 1383, 1336, 1325, 1392, 1341, 1329 |
| F3 |  | 3354, 3479, 3535, 3392, 3181, 3599, 3507, 3403, 3594, 3240, 3498, 3430, 3327, 3270, 3206, 3357 |
| F4 |  | 3451, 3593, 3736, 3447, 3434, 3687, 3776, 3610, 3275, 3370, 3506, 3405, 3741, 3328, 3297, 3422 |
| F5 |  | 6362, 6161, 6223, 6359, 6337, 6208, 6249, 6164, 6366, 6317, 6107, 6327, 6227, 6287, 6262, 6089 |
| F6 |  | 8550, 8413, 8603, 8537, 8449, 8402, 8447, 8497, 8553, 8643, 8628, 8521, 8484, 8411, 8419, 8619 |
| F7 |  | 9073, 9011, 9113, 9019, 9127, 9146, 9102, 9137, 9144, 9088, 9066, 9110, 9098, 9147, 9118, 9079 |
| F8 |  | 5202, 5326, 5332, 5314, 5468, 5216, 5169, 5456, 5394, 5412, 5315, 5298, 5192, 5210, 5600, 5349, 5280 |

**Table S2**

A list of amplification and sequencing primers used to generate full mtDNA genomes in this study. Primer sequences were slightly modified (by including degenerate bases) or were ordered unmodified from previous studies [7, 8].

| **Primer Name** | **Amp/Seq** | **Primer Sequence ( 5’ – 3’)** |
| --- | --- | --- |
| AV1F2 | Amp | AGGACTACGGCTTGAAAAGC |
| f1-3m | Seq | TGGTTCCTCGGTCAGGCACATCC |
| GLf2-3m | Amp | GGATTATCTTCCCCTCTTTAGT |
| AV3F | Seq | ATATACATGCAAGTATCCGC |
| AV4F | Amp | AAGACAGGTCAAGGTATAGC |
| AV5F | Seq | GCATCATGATTTAGCAAGAAC |
| AV6F | Amp | CAAGTATTGAAGGTGATGCC |
| AV7F | Seq | TAAGGGTTCGTTTGTTCAAC |
| f7-8m | Amp | ACTCCTCCTAGTACGAAAGGA |
| AV8F | Seq | AAATGCAAAAGGCTTAAGCC |
| AV9F | Amp | GCCCCATTTGACCTAACAGA |
| AV10F1 | Seq | TCACTATGATAAAGTGAACATAGA |
| f10-11m | Amp | CCCATTCCACTTCTGATTCCC |
| AV11F | Seq | CTAAACCAAACACAAACACGA |
| AV12F2 | Amp | GTTAAACTCTCTTAGTTTCTG |
| f12-13m | Seq | TCGTCATAATCTTCTTTATAG |
| AV13F | Amp | ATAGCATTCCCCCGCATAAA |
| AV14F2 | Seq | AAATGAGACCCCCCTATGCTA |
| AV15F2 | Amp | GTTAGTAAACCAATTACATAG |
| f15-17i | Amp | TTCATCACCACTCGACCAGGAGTG |
| AV16F | Amp | CACAGCTACATACCCATTGT |
| AV17F | Seq | ATAATCCAAGCCTACGTCTTC |
| f17-19i | Amp | CACTTCGTAGACATCATCTGA |
| AV18F | Seq | GCTCTTCTAGTATACTCATTACA |
| f18-20i | Amp | CAATGCTAAAGATCATCCTACCAA |
| AV19F | Seq | CACAACCTAAACCTTCTACAA |
| f19-21i | Amp | CCTCACCCTAGCCCTATGAGG |
| AV20F | Seq | CAAACAGACCTAAAATCCCTC |
| AV21F | Amp | TGCATCTGAGCTTTAAACCTC |
| f21-22m | Seq | CTAAGCATAGCATGACTAGCC |
| AV22F | Amp | CAGCAATAGAAGGCCCAACC |
| f22-23m | Seq | GAAAACCTAAACACCTCATAC |
| AV23F | Amp | TCACAAAAACTGCTGCCATC |
| f23-25i | Seq | ACAATACGGCTGACTCATCCGGAA |
| AV24F1 | Amp | GCCTCATTCTTCTTCATCTG |
| AV25F | Seq | CATTGTCTTGTAAACCAAAAAC |
| f25-1m | Amp | ACCCCATAATACGGCGAAGGATTA |
| AV1R2 | Seq | CATCTTGGCATCTTCAGTGCC |
| r2-3m | Amp | TGCTTAAGGTTAATTACTGCTG |
| AV2R | Seq | TCTTGGCATCTTCAGTGCCA |
| AV3R | Amp | TTAGAAAATGTAGCCCATTTCT |
| AV4R | Seq | CTCTTTTGCAACAGAGACGG |
| AV5R1 | Amp | GTTAGGATACCGCGGCCGTT |
| AV6R | Seq | CATAGATAGAAACCGACCTG |
| AV7R | Amp | GCTAGGGAGAGGATTTGAAC |
| r7-8m | Seq | TTTTCGTTCTACAAGTGTTAA |
| AV8R | Amp | GTATAATGTTGGCGTATTCG |
| AV9R | Seq | AACCATCATTTTCGGGGTAT |
| AV10R | Amp | GGATGAGAAGGCTAGGATTTT |
| r10-11m | Seq | GAGATGAGTATTGTTGACAGT |
| AV11R | Amp | GCGTTAGGCTGTAGTCCTTT |
| AV12R | Seq | GAGGAGTCAGAAGCTTATGTT |
| r12-13m | Amp | GTGATAAAGTTGATGGCTCCT |
| AV13R | Seq | ATAGTGGAAGTGGGCGACTA |
| AV14R | Amp | CTATGTAATTGGTTTACTAAC |
| AV15R2 | Amp | TGCATAGCTTCTTAATGGTTA |
| r15-17i | Amp | ACTATGTGGTAGGAGTGTGCTTGG |
| AV16R | Seq | ACTATGTGGTAGGAGTGTGC |
| AV17R | Amp | TGTTCATTGCTCTTCTCTGGG |
| r17-19i | Seq | TATGCCTGTTCAGAGGGTTAG |
| AV18R | Amp | AGGCTGTATATTGTGGTGTTA |
| r18-20i | Amp | AGGGAGGAGGTTAGTCCATGGGAG |
| AV19R | Amp | TGGGAGATTATGAGGATTATTG |
| r19-21i | Seq | CATTACTTTTACTTGGATTTG |
| AV20R | Amp | TACTTTTACTTGGATTTGCACC |
| AV21R2 | Seq | GCAACTACTATTGTGCTGGA |
| r21-22m | Seq | ATGAGGCCTAGTTGGCTTGA |
| AV22R | Seq | AGTTCTAGGGCTAGGATAATT |
| r22-23m | Amp | TAGGGGGTTAAAGTAGCCTA |
| AV23R | Seq | CCTGTGTTTCAGGTTTCCTT |
| r23-25i | Amp | AGTAGTTTATTTGAAAATACCAGC |
| AV24R | Seq | AATACCAGCTTTGGGAGCTG |
| AV25R | Amp | CTTTTCAAGCCGTAGTCCTT |
| r25-1m | Seq | ATGTCCTGTAACCATTCATAGT |

**Table S3**

A list of the primer sets used for pyrosequencing.

| **Primer Name** | **Sequence** | **Size (bp)** |
| --- | --- | --- |
| 95_NADH4L_pyro_F | CATCATTCGCCCTTGTACCTAT | 169 |
| 96_NADH4L_pyro_SEQ | CTGTGCCAGCTTCGCATG | - |
| 97_NADH4L_pyro_R | GGGCAAGAGTATGATTGTTGGT | - |
| 248_Cyt-b_pyro_F | GCCAACCTTCTTATCCTAACCTG | 67 |
| 249_Cyt-b_pyro_SEQ | TGATGATGAAGGGGTG | - |
| 250_Cyt-b_pyro_R | GGCCAATGATGATGAAGGG |  |

**Table S4**

Pyrosequencing results for chickens from the F_8_ and S41 generations included in this paper, indicating % of the wild type SNP ± 1σ.

| **Bird_ID** | **Generation** | **MT-ND4L** | **MT-ND4L**  **%G** | **CYTB** | **CYTB**  **%A** |
| --- | --- | --- | --- | --- | --- |
| 5168 | F8 | AA | 0.000 ± 0.00 | GG | 0.028 ± 0.02 |
| 5169 | F8 | AA | 0.000 ± 0.00 | GG | 0.052 ± 0.03 |
| 5456 | F8 | AA | 0.005 ± 0.01 | AG | 0.216 ± 0.02 |
| 2080 | S41 | AA | 0.015 ± 0.02 | AG | 0.298 ± 0.02 |
| 1945 | S41 | AA | 0.027 ± 0.01 | AA | 0.969 ± 0.03 |
| 2040 | S41 | AA | 0.039 ± 0.01 | AA | 0.991 ± 0.01 |
| 5349 | F8 | AA | 0.000 ± 0.00 | AA | 0.994 ± 0.01 |
| 1940 | S41 | AA | 0.016 ± 0.01 | AA | 0.993 ± 0.01 |
| 5315 | F8 | AA | 0.037 ± 0.00 | AA | 0.988 ± 0.00 |
| 5394 | F8 | AA | 0.000 ± 0.00 | AA | 0.988 ± 0.01 |
| 2026 | S41 | AG | 0.684 ± 0.04 | AA | 0.984 ± 0.00 |
| 1959 | S41 | AG | 0.129 ± 0.08 | AA | 0.986 ± 0.01 |
| 2068 | S41 | AG | 0.168 ± 0.01 | AA | 0.988 ± 0.00 |
| 2089 | S41 | AG | 0.437 ± 0.01 | AA | 0.991 ± 0.00 |
| 2130 | S41 | AG | 0.271 ± 0.01 | AA | 0.994 ± 0.01 |
| 5298 | F8 | AG | 0.091 ± 0.02 | AA | 0.996 ± 0.00 |
| 1934 | S41 | AG | 0.279 ± 0.02 | AA | 0.987 ± 0.00 |
| 1937 | S41 | AG | 0.652 ± 0.03 | - | - |
| 1740 | S41 | GG | 0.988 ± 0.01 | - | - |
| 1690 | S41 | GG | 0.992 ± 0.01 | AA | 0.945 ± 0.04 |
| 1653 | S41 | GG | 0.991 ± 0.01 | AA | 0.968 ± 0.03 |
| 1967 | S41 | GG | 0.989 ± 0.01 | AA | 0.978 ± 0.01 |
| 1897 | S41 | GG | 0.991 ± 0.02 | AA | 0.978 ± 0.01 |
| 1932 | S41 | GG | 1.000 ± 0.00 | AA | 0.978 ± 0.02 |
| 1812 | S41 | GG | 0.983 ± 0.00 | AA | 0.979 ± 0.03 |
| 1774 | S41 | GG | 0.997 ± 0.00 | AA | 0.979 ± 0.00 |
| 1670 | S41 | GG | 0.990 ± 0.02 | AA | 0.980 ± 0.00 |
| 1925 | S41 | GG | 0.994 ± 0.00 | AA | 0.980 ± 0.01 |
| 2100 | S41 | GG | 0.988 ± 0.02 | AA | 0.981 ± 0.00 |
| 1736 | S41 | GG | 0.999 ± 0.00 | AA | 0.981 ± 0.01 |
| 1728 | S41 | GG | 0.996 ± 0.01 | AA | 0.982 ± 0.02 |
| 1879 | S41 | GG | 0.997 ± 0.01 | AA | 0.982 ± 0.00 |
| 2104 | S41 | GG | 0.993 ± 0.01 | AA | 0.984 ± 0.02 |
| 1654 | S41 | GG | 0.995 ± 0.01 | AA | 0.985 ± 0.01 |
| 1953 | S41 | GG | 0.990 ± 0.01 | AA | 0.985 ± 0.01 |
| 2029 | S41 | GG | 0.979 ± 0.02 | AA | 0.986 ± 0.02 |
| 2109 | S41 | GG | 0.981 ± 0.02 | AA | 0.986 ± 0.00 |
| 1712 | S41 | GG | 0.986 ± 0.01 | AA | 0.987 ± 0.01 |
| 1832 | S41 | GG | 1.000 ± 0.00 | AA | 0.987 ± 0.01 |
| 2012 | S41 | GG | 0.992 ± 0.01 | AA | 0.987 ± 0.00 |
| 1655 | S41 | GG | 0.988 ± 0.01 | AA | 0.988 ± 0.01 |
| 2132 | S41 | GG | 1.000 ± 0.00 | AA | 0.988 ± 0.00 |
| 1678 | S41 | GG | 1.000 ± 0.00 | AA | 0.989 ± 0.01 |
| 1720 | S41 | GG | 0.982 ± 0.01 | AA | 0.989 ± 0.01 |
| 1680 | S41 | GG | 0.987 ± 0.01 | AA | 0.989 ± 0.00 |
| 1871 | S41 | GG | 0.991 ± 0.01 | AA | 0.989 ± 0.01 |
| 1896 | S41 | GG | 1.000 ± 0.00 | AA | 0.991 ± 0.01 |
| 1926 | S41 | GG | 0.994 ± 0.01 | AA | 0.991 ± 0.00 |
| 1784 | S41 | GG | 0.988 ± 0.01 | AA | 0.991 ± 0.01 |
| 1674 | S41 | GG | 0.984 ± 0.01 | AA | 0.992 ± 0.01 |
| 1833 | S41 | GG | 0.996 ± 0.00 | AA | 0.992 ± 0.01 |
| 1927 | S41 | GG | 0.970 ± 0.00 | AA | 0.992 ± 0.01 |
| 1890 | S41 | GG | 0.999 ± 0.00 | AA | 0.992 ± 0.01 |
| 2048 | S41 | GG | 1.000 ± 0.00 | AA | 0.992 ± 0.01 |
| 1757 | S41 | GG | 0.991 ± 0.01 | AA | 0.993 ± 0.01 |
| 1786 | S41 | GG | 1.000 ± 0.00 | AA | 0.993 ± 0.01 |
| 2108 | S41 | GG | 0.984 ± 0.00 | AA | 0.993 ± 0.01 |
| 1819 | S41 | GG | 0.988 ± 0.01 | AA | 0.993 ± 0.01 |
| 1972 | S41 | GG | 1.000 ± 0.00 | AA | 0.993 ± 0.01 |
| 1992 | S41 | GG | 0.986 ± 0.01 | AA | 0.994 ± 0.01 |
| 1988 | S41 | GG | 0.986 ± 0.01 | AA | 0.994 ± 0.01 |
| 2064 | S41 | GG | 0.987 ± 0.01 | AA | 0.994 ± 0.01 |
| 1822 | S41 | GG | 0.981 ± 0.02 | AA | 0.994 ± 0.01 |
| 1844 | S41 | GG | 0.995 ± 0.00 | AA | 0.994 ± 0.01 |
| 1815 | S41 | GG | 0.987 ± 0.02 | AA | 0.995 ± 0.00 |
| 1997 | S41 | GG | 0.995 ± 0.01 | AA | 0.995 ± 0.00 |
| 5192 | F8 | GG | 0.979 ± 0.02 | AA | 0.961 ± 0.04 |
| 5280 | F8 | GG | 0.986 ± 0.01 | AA | 0.986 ± 0.02 |
| 5206 | F8 | GG | 0.968 ± 0.03 | AA | 0.989 ± 0.00 |
| 5185 | F8 | GG | 0.982 ± 0.02 | AA | 0.989 ± 0.00 |
| 5314 | F8 | GG | 0.988 ± 0.01 | AA | 0.989 ± 0.01 |
| 5600 | F8 | GG | 0.983 ± 0.02 | AA | 0.990 ± 0.00 |
| 5210 | F8 | GG | 0.998 ± 0.00 | AA | 0.990 ± 0.00 |
| 5332 | F8 | GG | 0.977 ± 0.02 | AA | 0.991 ± 0.00 |
| 5468 | F8 | GG | 0.976 ± 0.02 | AA | 0.993 ± 0.01 |
| 5216 | F8 | GG | 0.968 ± 0.01 | AA | 0.994 ± 0.01 |
| 5202 | F8 | GG | 0.981 ± 0.01 | AA | 0.997 ± 0.01 |
| 5326 | F8 | GG | 0.979 ± 0.02 | AA | 0.997 ± 0.01 |

**Table S5.** Effect of mtDNA genotype, scored as the percentage of the mutant sequence, as regards *de novo* mutations in *ND4L* and *CYTB* on body-weight at various ages in the F_8_ generation (n=378) from an intercross between Virginia High-Weight and Low-Weight chickens. The mtDNA genotype was determined by pyrosequencing or for many of the wild type birds deduced based on the maternal lineage.

| Locus | | | | | | | | | |  |
| --- | --- | --- | --- | --- | --- | --- | --- | --- | --- | --- |
|  | *NAD4HL* | | |  | | *CYTB* | | | |  |
| Weight^1^ |  | Effect^2^±SE | *P*^3^ | |  | |  | Effect^2^±SE | *P*^3^ | |
| Hatch |  | -0.3±0.7 | 0.72 | |  | |  | -0.9±0.9 | 0.36 | |
| 14 days |  | 3.3±3.6 | 0.36 | |  | |  | 1.9±4.6 | 0.68 | |
| 28 days |  | 6.5±10.0 | 0.51 | |  | |  | 1.4±12.5 | 0.91 | |
| 42 days |  | 17.4±19.8 | 0.38 | |  | |  | 7.2±24.8 | 0.77 | |
| 56 days |  | 35.1±30.6 | 0.25 | |  | |  | 19.2±38.5 | 0.62 | |
| 70 days |  | 69.5±44.1 | 0.12 | |  | |  | 34.9±55.5 | 0.53 | |

^1^Body-weight (g) at given age; ^2^Effect (g) of mtDNA genotype at the tested locus ± standard error of the mean; ^3^Significance value for the effect of mtDNA genotype on body-weight at the given age estimated using linear regression with the model Y = S + H + E, where S=sex of the bird, H=percentage of mutant sequence on scale 0-1 (see Table S4) where 1 is the wild-type, and E=residual error
